# Plasma cell treatment device *Plasma-on-Chip*: Monitoring plasma-generated reactive species in microwells

**DOI:** 10.1038/srep41953

**Published:** 2017-02-08

**Authors:** Jun-Seok Oh, Shinya Kojima, Minoru Sasaki, Akimitsu Hatta, Shinya Kumagai

**Affiliations:** 1 Department of Electronic and Photonic Systems Engineering, Kochi University of Technology, Kami, Kochi, 782-8502 Japan; 2 Center for Nanotechnology, Research Institute of Kochi University of Technology, Kami, Kochi, 782-8502 Japan; 3 Department of Advanced Science Engineering, Toyota Technological Institute, Nagoya, 468-8511 Japan

## Abstract

We have developed a plasma cell treatment device called *Plasma-on-Chip* that enables the real-time monitoring of a single cell culture during plasma treatment. The device consists of three parts: 1) microwells for cell culture, 2) a microplasma device for generating reactive oxygen and nitrogen species (RONS) for use in cell treatment, and 3) through-holes (microchannels) that connect each microwell with the microplasma region for RONS delivery. Here, we analysed the delivery of the RONS to the liquid culture medium stored in the microwells. We developed a simple experimental set-up using a microdevice and applied *in situ* ultraviolet absorption spectroscopy with high sensitivity for detecting RONS in liquid. The plasma-generated RONS were delivered into the liquid culture medium via the through-holes fabricated into the microdevice. The RONS concentrations were on the order of 10–100 μM depending on the size of the through-holes. In contrast, we found that the amount of dissolved oxygen was almost constant. To investigate the process of RONS generation, we numerically analysed the gas flow in the through-holes. We suggest that the circulating gas flow in the through-holes promotes the interaction between the plasma (ionised gas) and the liquid, resulting in enhanced RONS concentrations.

In medicine, the term “plasma” is used to describe the liquid component of blood, whereas in physics and chemistry, the term refers to one of the four fundamental states of matter. Plasma (hereafter used in the latter sense) consists of positively and negatively charged particles as well as neutral particles. Since plasma has shown potential applications in the medical field, a research sub-field called “plasma medicine” has emerged^1^,^2^,^3^,^4^,^5^,^6^,^7^,^8^,^9^. Over the last 20 years, many researchers have explored the potential applications of plasma in the biomedical field^10^,^11^,^12^,^13^,^14^,^15^,^16^,^17^,^18^,^19^,^20^. Non-thermal atmospheric pressure plasmas (NTAPPs) have been used for biomedical applications. During NTAPP treatment, biological samples are not damaged. The word “non-thermal” in NTAPPs indicates “cold” or “room temperature”. NTAPPs efficiently produce reactive oxygen species (ROS) and/or reactive nitrogen species (RNS), which are often collectively described as reactive oxygen and nitrogen species (RONS)^21^,^22^. The behaviour of RONS accounts for the biomedical effects of plasma and is therefore important in plasma medicine^20^,^21^,^22^,^23^,^24^,^25^.

ROS are generated within plant and animal cells, including human cells, and are known to regulate key biochemical pathways that are vital to the maintenance of normal physiological functions and the fight against diseases^26^. It is important to note that plasma can generate ROS, which can aid in healing chronic wounds, curing bacterial infections, and killing cancer cells^9^,^27^,^28^,^29^.

During plasma treatment, a broad area of tissue is exposed to the plasma. It is readily understood that the plasma affects cells in the vicinity of the treated area. Figure 1 illustrates what occurs when the plasma irradiation area is reduced to a size as small as a cell (plasma ≒ cell) or smaller (plasma < cell)^30^,^31^,^32^,^33^,^34^. In these cases, we expect that the plasma treatment activates or inactivates biological reactions at the single-cell level.

We have developed a plasma cell culture device that enables plasma treatment at the single-cell level (Fig. 2)^32^,^33^,^34^. The device is referred to as “*Plasma-on-Chip*” and is used to analyse the interactions between the plasma and cells in real time. The *Plasma-on-Chip* device consists of three parts that are fabricated together in a silicon (Si) chip. Microwells are fabricated for cell culture, and on the backside of each microwell, there is a microplasma source that consists of a pair of microelectrodes. Between the microwell and microplasma regions are microscopic through-holes that connect the two regions. When liquid containing cells is poured into the microwell, a gas-liquid interface forms from the surface tension, resulting in no leakage from the microwell. The RONS are generated at the microplasma source on the chip^5^,^35^. The through-holes function as microchannels for RONS delivery so that the reactive species from the plasma are delivered to the cells via the through-hole(s), gas-liquid interface and a thin layer of the liquid. If the size of the through-hole is reduced to smaller than that of a cell, the cell is locally treated with plasma, which can selectively activate or inactivate the specific functions of the cell. Thus far, we have reported the inactivation of *Chlorella* cells with the *Plasma-on-Chip* device^32^,^34^

The *Plasma-on-Chip* device provides advantages to cell analysis. Suppose that a cell population is analysed after plasma irradiation. In general, the cells seem to be morphologically identical in a population. However, a recent study revealed that the gene expressions of individual cells vary according to environmental conditions, even in a homogeneous population^36^. Therefore, single-cell analysis is important and has been studied thus far^37^,^38^,^39^. If plasma irradiation induces a biological reaction in a few cells, a population-averaged analysis conceals the characteristics of individual cells. Using the *Plasma-on-Chip* device, we can seed one cell in one microwell (Fig. 2) and subsequently monitor and analyse the responses of the cell without concealment by the population-averaged measurement. The *Plasma-on-Chip* device enables one to directly discuss the cause and effect between the plasma irradiation and a cell. There are also many other potential applications of the *Plasma-on-Chip* device. For example, since plasma irradiation has been used in cell permeabilisation, the *Plasma-on-Chip* device can be used for gene transfection at the single-cell level^40^,^41^,^42^.

To analyse the plasma-cell interaction in more detail, we should know the behaviour of the reactive species during treatment. In the gas phase, optical emission spectroscopic studies showed that the plasma can generate elements to form RONS, including excited O* atoms, excited N_2_ molecules, and OH radicals^32^,^34^,^43^. Ionic species were also identified by ambient molecular beam mass spectrometry^44^,^45^,^46^. Understanding the behaviour of the reactive species in the liquid phase is also necessary. Various RONS with long and short lifetimes have been discussed widely in the plasma medicine field^21^,^22^,^47^,^48^. However, detecting the RONS generated by the *Plasma-on-Chip* device is difficult because the microwell volume (100 μm × 100 μm × 200 μm) is extremely small (approximately a few pL). Thus, only an exceedingly small volume of liquid media is available for analysis, and it has been impossible to directly analyse such a small sample.

To address this challenge, we developed a simple experimental set-up for analysing the behaviour of the plasma-generated RONS in microwells. We used ultraviolet (UV) absorption spectroscopy associated with high-quality quartz cuvettes. Measurements of the absorption spectra in the UV range (190–340 nm) enabled us to detect very low concentrations of RONS in deionised (DI) water^49^,^50^,^51^,^52^. Using the UV absorption spectroscopy data, we found that the revised detection limit of the RONS in liquid was on the order of fM.

## Results

### A simple experimental set-up for the demonstration of reactive species delivered via through-holes

To analyse the delivery of plasma-generated reactive species to the microwells, a Si chip device with through-holes and a NTAPP jet (NTAPPJ) source were used (Fig. 3). The plasma jet generated the reactive species in the gas phase over the Si chip, and the flow of the plasma jet delivered the reactive species into the DI water underneath the Si chip via the through-holes. Thus, we demonstrated the delivery of the reactive species with this set-up.

Arrays of through-holes were fabricated in a 200-μm-thick Si substrate (22 mm × 22 mm) by a deep reactive ion etching technique (etching depth: 200 μm)^32^. Three different sizes (50 μm × 50 μm, 100 μm × 100 μm, and 200 μm × 200 μm) of through-hole arrays were created, with distances between the through-holes of 150 μm, 300 μm, and 600 μm, respectively. Considering the diameter of the plasma jet (ϕ800 μm), the plasma irradiation (or the core of the gas flow) covered 8–13, 2–4, and 0–1 through-holes for a 50-μm, 100-μm, and 200-μm device, respectively (Supplementary information, Fig. S1). Scanning electron microscope (SEM) images of 200 μm × 200 μm through-holes are shown in Fig. 3. The Si chip with through-holes was placed on top of a quartz cuvette filled with DI water (~4.1 mL). The Si chips were large enough to cover the open area of the cuvette (10 mm × 10 mm). We carefully prepared the experimental set-up for removing the air pocket between the Si chip and the water surface. The experimental section provides more detail on how we removed the air pocket between the Si chip and the cuvette. The exposure of the NTAPPJ or gas flow, however, created a very thin air pocket between the Si chip and the water surface. The depth of the air pocket (~several hundred μm) was estimated from the weight change of the water in the cuvette.

**Figure 2:**
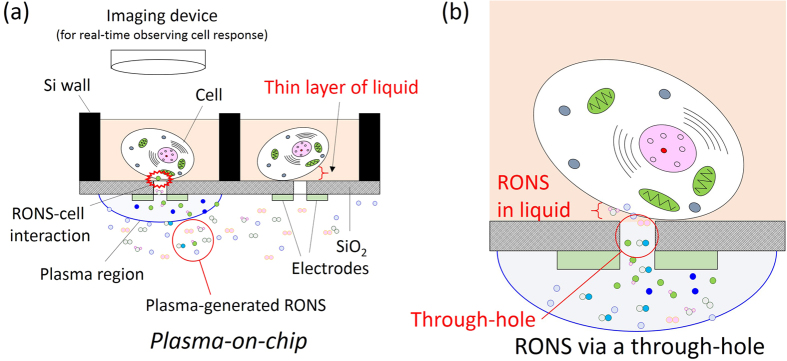
Conceptual illustration of the *Plasma-on-Chip* device for the real-time monitoring of the interactions between the plasma-generated RONS and single cells. (**a**) Magnified illustration of the area around a through-hole. (**b**) The through-hole plays the role of a microchannel for the delivery of plasma-generated RONS. The plasma-generated RONS pass through the thin layer of liquid and reach the cell.

**Figure 3:**
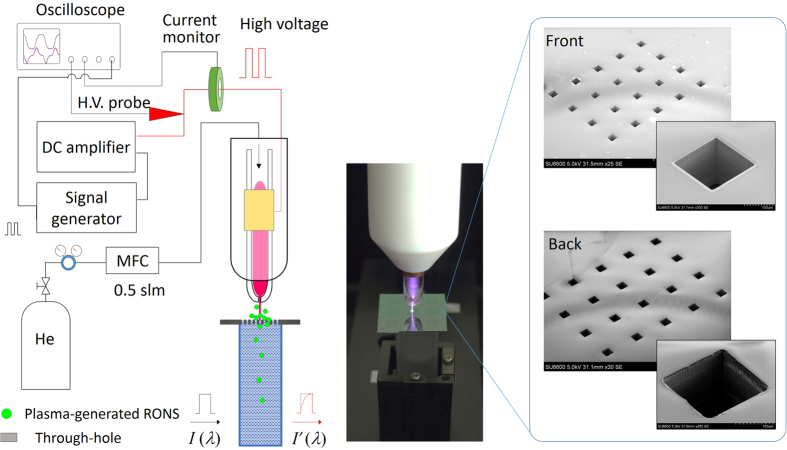
Experimental set-up employed for *in situ* UV absorption spectroscopy to monitor the delivery of RONS via a device containing microscopic through-holes to the DI water during NTAPPJ irradiation. The NTAPPJ is irradiated onto a cluster of through-holes. The reactive species that passed through the through-holes are detected by UV absorption spectroscopy. The photograph (middle) shows the UV measurement, and the scanning electron microscope (SEM) images show the front and back sides of the through-holes.

A helium gas flow of 0.5 standard litres per min (slpm) was fed into a glass tube around which was wound a copper electrode. The electrode was connected to a direct-current high-voltage amplifier and a pulse generator. High-voltage square pulses (with a fixed voltage of 7 kV_p-p_, peak to peak, and a fixed frequency of 10 kHz) were applied to the electrode. For comparison, the typical operation conditions of the *Plasma-on-Chip* device were 0.5–1.0 slpm of He and 0.5–1.0 kV at a frequency of 1–10 kHz^33^,^34^. In the cases involving the *Plasma-on-Chip*, or those using NTAPPJ and the through-hole devices, reactive species were generated by the interactions between the emerging He plasma and the ambient air. When the plasma-generated reactive species passed through the through-holes and dissolved in DI water, we detected a UV absorption signal. Using our simply designed experimental set-up, we simulated the delivery of RONS to the microwells of the *Plasma-on-Chip* device (Fig. 2b).

### Detection of reactive species in DI water

**Figure 4:**
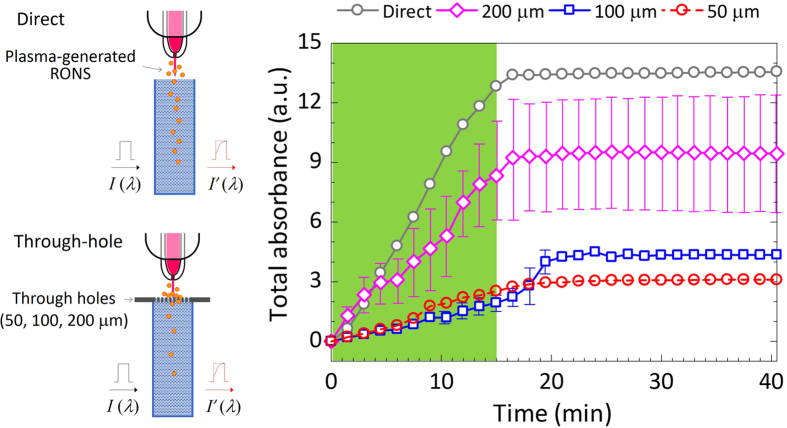
Time-dependent changes in the total UV absorbance between 190–340 nm. A cluster of through-holes was irradiated with NTAPPJ. Curves of the total absorbance are measured under direct and indirect NTAPPJ irradiation using the through-hole devices. The NTAPPJ was applied for 15 min. After 15 min, the plasma generation and He gas flow were stopped. The size of the through-hole device was 22 mm × 22 mm and 200 μm in thickness. The through-hole device was set on a quartz cuvette (10 mm × 10 mm × 41 mm). Each measurement was carried out three times, and the mean values are shown with error bars. If a plot has no error bars, it means that the error range is small.

*In situ* UV absorption spectroscopy was conducted throughout the periods of plasma generation (15 min) and after plasma treatment (25 min), which occurred when the plasma generation and He gas flow were stopped. Owing to the scanning speed of the UV absorption spectroscope, it took one minute and a half to detect the plasma-generated reactive species at the measuring point in the cuvette (3 cm below the DI water surface). The time dependencies of the total absorbance are shown in Fig. 4. After the detection delay, responses were observed in the total absorbance. The total absorbance increased with the NTAPPJ irradiation time. Without the NTAPPJ irradiation, the intensity was almost constant. Remarkably, the total absorbance clearly depended on the size of the through-holes.

**Figure 5:**
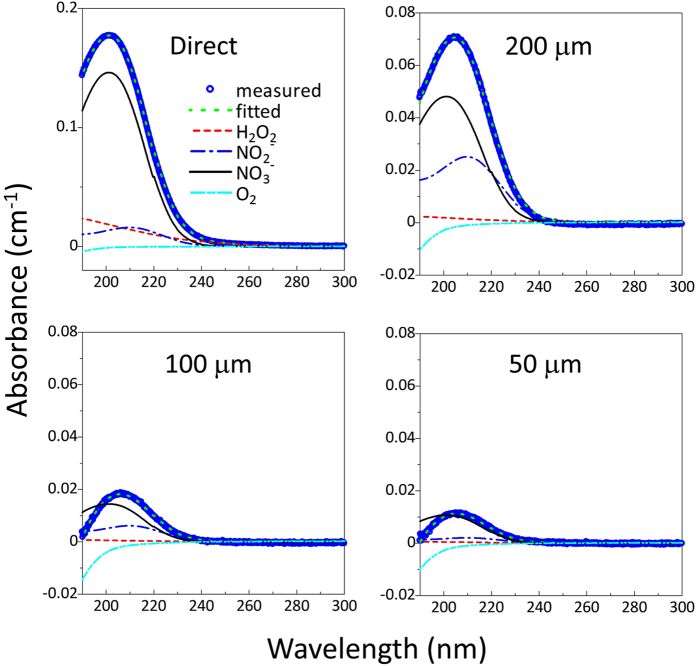
Curve-fitted results of the UV absorption spectra of the plasma-irradiated DI water. The measurements were conducted at the time point of 40.5 min for direct and indirect plasma-irradiation using three different-sized through-holes (200 μm × 200 μm, 100 μm × 100 μm, and 50 μm × 50 μm). The absorption spectra were acquired at 40 min. The spectra were decomposed using the reference data of H_2_O_2_, NO_2_^−^, NO_3_^−^, and dissolved O_2_. The size of the through-hole device was 22 mm × 22 mm and 200 μm in thickness. The through-hole device was set on a quartz cuvette (10 mm × 10 mm × 41 mm).

The absorption profiles of the plasma-irradiated DI water were curve-fitted using an automated program developed at Kochi University of Technology^49^,^52^. The curve-fitting was based on a database of absorption spectra of reference solutions with known concentrations^49^,^50^,^52^. We used the databases of three reference solutions, hydrogen peroxide (H_2_O_2_), sodium nitrite (NaNO_2_), and nitric acid (HNO_3_), to calculate the concentrations of the plasma-generated species: H_2_O_2_, nitrite (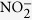) and nitrate (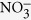). We also used the dissolved O_2_ in the DI water concentration database. The curve-fitting results at 40.5 min are shown in Fig. 5. Each total absorbance curve was broken down into four curves of H_2_O_2_, 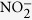, 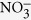, and dissolved O_2_. The absorption intensities of the four components decreased when the through-hole device was used. The UV absorption signal of H_2_O_2_ was smaller than those of 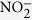 and 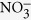 in the measured wavelength range of 190–340 nm. Note that under the same concentration conditions reported in our previous study^52^, the absorption signal of H_2_O_2_ was 10 and 20 times smaller than those of 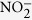 and 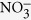, respectively.

### Time-dependent RONS and O_2_

**Figure 6:**
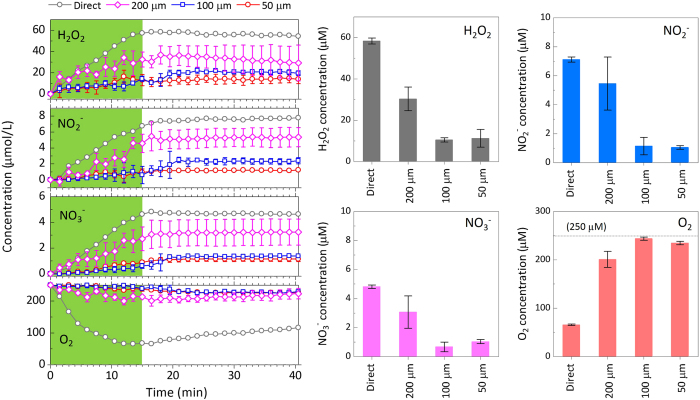
Time-dependent changes in the concentrations of H_2_O, 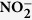, 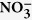 and O_2_ in DI water treated directly or indirectly (using the through-hole devices) with NTAPPJ. A cluster of through-holes was irradiated with NTAPPJ for 15 min (green shaded area). The size of the through-hole device was 22 mm × 22 mm and 200 μm in thickness. The through-hole device was set on a quartz cuvette (10 mm × 10 mm × 41 mm). Each measurement was carried out three times, and the mean values are shown with error bars. If a plot has no error bars, it means that the error range is small. The bar graphs show the mean concentrations at the time point of 16.5 min. The dotted line shown in the graph of the O_2_ concentration corresponds to the initial O_2_ concentration before the plasma irradiation (250 μM).

The concentrations of H_2_O_2_, 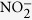, 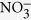, and dissolved O_2_ were plotted as functions of time (Fig. 6). Under ambient conditions (atmospheric pressure and room temperature), 250 μM of oxygen molecules are dissolved in DI water. The concentrations of H_2_O_2_, 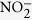, and 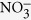 increased with the NTAPPJ irradiation time. After 15 min, the NTAPPJ was turned off and the gas feed was stopped. The concentrations of H_2_O_2_, 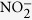, and 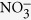 were then at almost the same level. However, the dissolved O_2_ concentration exhibited different behaviour under the direct irradiation conditions. During the NTAPPJ irradiation, the dissolved O_2_ concentration decreased in all situations (direct and through the three different-sized through-holes). After the plasma treatment, the O_2_ concentrations recovered in the direct irradiation case (without the through-hole device on the water surface), but they remained at almost the same level or decreased slightly in the indirect irradiation case (when the through-hole device was used).

In the UV absorption spectroscopy system, it takes one minute and a half for scanning the UV range. Since the duration of the plasma irradiation was 15 min, the time delay led the last signals of the RONS generation in the UV absorption curves to be received at 16.5 min. Therefore, the RONS generation signals were included in the results up to 16.5 min. The mean concentrations of RONS and dissolved oxygen at 16.5 min are also shown in Fig. 6. Smaller RONS concentrations were detected for the 50-μm and 100-μm through-hole devices than for the 200-μm device.

### Concentration of RONS delivered via one through-hole per discharge period

Since the *Plasma-on-Chip* device has one through-hole, as shown in Fig. 2, it was useful to estimate the concentrations of long-lived species (H_2_O_2_, 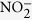, and 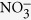) via one through-hole per pulse of the plasma-generation voltage signals. For the estimation, we used the results of 50 μm × 50 μm through-holes because the total absorbance curve of the 50 μm × 50 μm through-hole device had the smallest error bars among the three devices, as shown in Fig. 4. From the results shown in Fig. 6, the concentrations of H_2_O_2_, 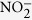 and 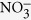 in 4.1 mL of DI water were 13.9 μM, 1.23 μM and 1.14 μM, respectively, at the time point of 16.5 min. In the experimental geometry, the plasma jet covered 8–13 through-holes. Considering these concentration and experimental configuration results, 0.12–0.15 aM (10^−18^ M) of H_2_O_2_ was delivered to the water via one through-hole per discharge period (100 μs). Additionally, 0.011–0.014 aM of 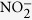 and 0.010–0.016 aM of 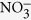 were delivered via the through-hole.

### Gas flow in through-holes

**Figure 7:**
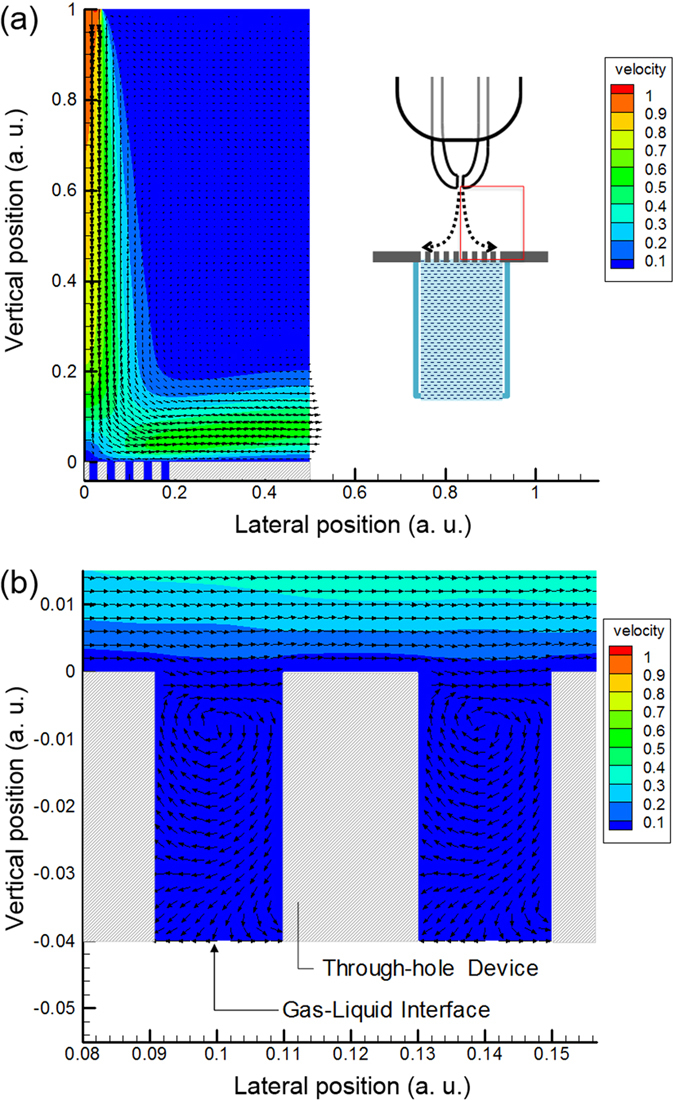
(**a**) Simulation results of gas flow in the vicinity of the through-hole structures. The inset is a schematic drawing of the plasma irradiation using a through-hole device. The area indicated by the red box in the inset is the simulated area of irradiation. The Reynolds number is 1000. The vertical position is normalised to the distance between the orifice and the through-hole device. The arrow directions and lengths indicate the flow directions and velocity magnitude, respectively. (**b**) Magnified image of gas flows in the through-hole area. The arrows have a constant length to visualise the small flows.

The gas flow under the indirect plasma irradiation (with through-holes) was numerically analysed using a simple model (Supplementary information, Fig. S2). Figure 7 shows the result for a Reynolds number of 1000. Each bottom of a through-hole device corresponded to the gas-liquid interface. The arrows indicate the flow direction, and their lengths correspond to the magnitude of the gas flow velocities. The gas flow from a NTAPPJ orifice collides with the surface of the through-hole device, causing its flow direction to change to the horizontal^53^. Consequently, the gas flow moves along the surface of the through-hole device but does not penetrate deep into the through-holes. In the through-holes, the flow magnitudes are very small (Fig. 7a); to show these small flows, the arrows were replotted with a constant length (Fig. 7b), which showed that circulating flows had formed.

### Vaporisation

**Figure 8:**
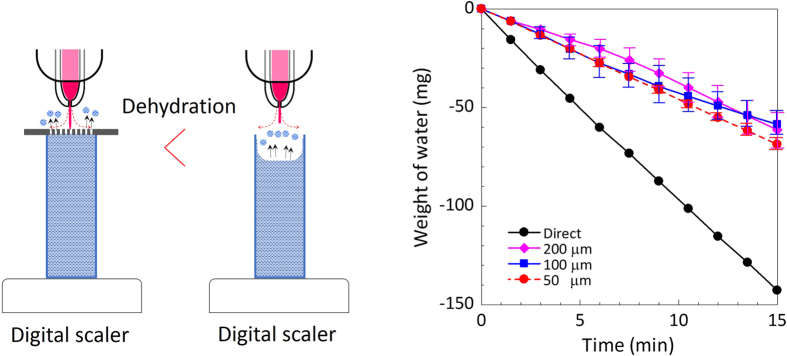
Weight changes of DI water through vaporisation induced by He gas flow. Using a digital scale, the weight changes were measured for direct (without through-hole) and indirect (with 200, 100, and 50 μm through-hole devices) plasma irradiation.

The gas flow of He can reduce the water volume in a cuvette through vaporisation. The vaporised water molecules migrate to the ambient air, probably increasing the local humidity and interacting with the emerging plasma species. We measured the weight changes of the DI water in a cuvette with or without a through-hole device using a digital scale (ViBRA, Shinko Denshi Co., Ltd., Tokyo, Japan) (Fig. 8). High vaporisation (a weight change of 143 mg after 15 min) was found for the direct plasma irradiation condition, while lower vaporisation (~70 mg) was observed when the through-hole devices were used. This result partially explains the higher concentrations of H_2_O_2_ under the direct plasma irradiation (Fig. 6).

## Discussion

In this study, the delivery process of plasma-generated reactive species was modelled using a through-hole device and NTAPPJ source. In general, the largest concentration changes of the reactive species were observed for the direct NTAPPJ irradiation. However, we note that the RONS concentrations resulting from indirect irradiation were much larger than those that were simply estimated from the areas over which the plasma irradiated the water surface.

The area of the NTAPPJ covering the through-holes defines the area over which the plasma irradiates the water surface. We estimated this area from the experimental geometry (see the details in the experimental section of NTAPPJ); the estimated values were 0.50 mm^2^, 0.04 mm^2^, 0.04 mm^2^, and 0.03 mm^2^ for the direct case and for the 200-μm, 100-μm, and 50-μm through-hole devices, respectively. The ratio of the water surface area exposed to plasma irradiation was 1 : 0.08 : 0.08 : 0.06. When we considered the plasma irradiation water surface area ratio (1 : 0.08 : 0.08 : 0.06), the RONS concentrations with indirect irradiation using the through-hole device were approximately 10% of that under direct NTAPPJ irradiation.

We next calculated the RONS concentration ratio using the results shown in Fig. 6. The total concentrations of RONS (sums of H_2_O_2_, 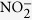, and 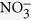 concentrations) at the time point of 16.5 min were 70.3 μM, 38.8 μM, 12.3 μM, and 13.3 μM for the direct, 200-μm, 100-μm, and 50-μm through-hole devices, respectively. The ratio of the total RONS concentrations was reduced from 70.3 : 38.8 : 12.3 : 13.3 to 1 : 0.55 : 0.18 : 0.19. The RONS concentrations of the indirect irradiations were 55% (200-μm), 18% (100-μm), and 19% (50-μm) of the values for the direct irradiation; these values were each larger than the 10% value estimated from the plasma-irradiation area. We speculated that local flow induced by the periodic through-holes enhanced the contact between the RONS and DI water^54^.

Regarding the concentrations of H_2_O_2_, 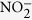 and 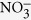, the behaviours for the 50 μm × 50 μm and 100 μm × 100 μm through-holes were almost the same. However, the 200 μm × 200 μm through-hole behaviour was different, indicating that the reactive species dissipated during the delivery via the through-holes. Since the thickness of each Si substrate was 200 μm, the aspect ratios for the 50 μm × 50 μm, 100 μm × 100 μm, and 200 μm × 200 μm through-holes were calculated as 4, 2, and 1, respectively. We suggest that the RONS collision loss with the through-hole wall emerged at a critical point, specifically, at an aspect ratio of 2. Beyond the critical point, the RONS delivered to the water decreased. At atmospheric pressure, the width of a plasma sheath can vary between 100–350 μm, depending on the operating parameters of the discharge^44^,^55^. Our observations support our assertion that the plasma can directly contact the water surface, resulting in an increased RONS concentration.

Each RONS behaviour is time-dependent. The concentrations of 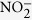 and 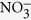 increased with the NTAPPJ irradiation time. After the irradiation was stopped, those concentrations remained at an almost constant level. As the concentration trends corresponded well to the NTAPPJ irradiation and since the nitrogen was assumed to be derived from the ambient air, we suggest that both 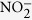 and 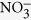 were derived from the chemical species generated in the gas phase.

For H_2_O_2_, the trend was basically similar to those of 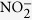 and 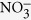. Regarding the generation of H_2_O_2_ in our experimental system, we must consider both the source of the hydroxyl radical and the behaviour of the water vapour. For example, H_2_O_2_ can be generated in an interaction between He plasma and water in humid air as per the following equations^54^:


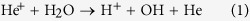



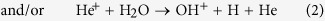



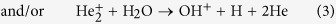



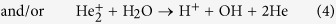



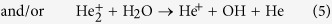



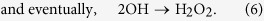


Therefore, the amount of water vapour could affect the generation of H_2_O_2_.

Interestingly, we observed different behaviour for the dissolved oxygen. Under the direct NTAPPJ irradiation of DI water, the dissolved oxygen concentration decreased, which indicated the deoxygenation (sparging) of the DI water. The deoxygenation resulted from the purging effect of the He gas flow during the NTAPPJ irradiation. Under ambient conditions, 250 μM of oxygen molecules were dissolved in DI water at the initial state (before the plasma irradiation). After 15 min of NTAPPJ irradiation, the dissolved oxygen concentration decreased to 70 μM. It is possible that this decrease in the O_2_ concentration affected the cells’ activities^56^. Once the plasma irradiation and He gas feed were turned off, the gas purge stopped, and the ambient gas again dissolved in the DI water. The dissolved O_2_ concentration then recovered to 120 μM after 25 min. In contrast, under indirect irradiation with the through-holes, the deoxygenation was much lower than that under direct irradiation. Introducing the through-hole device decreased the amount of gas blowing over the liquid surface, resulting in a reduced gas purge effect.

A numerical analysis of the gas dynamics showed that the gas flow from the orifice did not reach the liquid surface directly. This indirect gas flow may be one of the reasons for the low deoxygenation. Furthermore, we note that there were circulating flows in the through-holes (Fig. 7). The circulating flow can promote the interaction between the gas and liquid. As a result, RONS are generated and dissolve into the DI water. Thus, the amount of RONS was higher than the amount estimated from the total through-hole areas.

The measurements of the water vaporisation indicated that a large amount of water vapour was fed to the plasma region under the direct NTAPPJ irradiation of the liquid surface. Using the through-hole device, the amount of water vapour generated by the NTAPPJ irradiation was reduced. This reduction partially explains the low H_2_O_2_ concentration. As shown in Fig. 8, it is interesting that the vaporisation was slightly enhanced for the smaller through-hole sizes. This enhancement may have resulted from the combination of two effects: the capillary force generated at the through-holes and the increased hydrophilicity of the plasma-irradiated through-hole device surface (SiO_2_). Both effects can raise the level of the DI water in the through-hole towards the outside. As a result, the interaction between the NTAPPJ and the DI water increased the amounts of RONS. Further investigations into this phenomenon are underway.

In this study, we successfully demonstrated the delivery of plasma-generated RONS into DI water via microscopic through-holes using a simple experiment set-up with NTAPPJ, a through-hole device, and *in situ* UV absorption spectroscopy. From the analysis of the UV absorption spectra, increases in the H_2_O_2_, 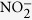, and 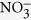 concentrations and a decrease in the dissolved O_2_ were observed. The time-dependent changes in the concentrations of H_2_O_2_, 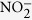, and 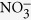 showed similar trends. The concentrations increased during the NTAPPJ irradiation, but they were lower than those generated by direct NTAPPJ irradiation.

The UV absorption spectroscopy method developed here will be applied to the *Plasma-on-Chip* device. Since the *Plasma-on-Chip* device has one through-hole in the microwell, as shown in Fig. 2, we should consider the size of the plasma irradiation area in discussing the generation and delivery processes of RONS.

The RONS measured in this study, H_2_O_2_, 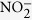, and 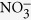, have long lifetimes. It would also be interesting to analyse the behaviours of short-lived ROS (e.g., O, OH, and O_2_^−^) in liquid because those RONS have high redox potentials that can affect cells’ activities. Further investigations are needed to obtain a complete understanding of the situation of the cell(s) in the microwells.

## Experimental Section

### Materials

Non-doped, 200-μm-thick double-side polished Si wafers (Yamanaka Hutech Corp., Kyoto, Japan) were used. Both sides were thermally oxidised to form 3-μm SiO_2_ layers.

### Fabrication of through-hole devices

Three Si chip were prepared, and photoresist films were formed on each one. Photolithography was used to make the through-hole patterns on the chips. The photoresist patterns of the through-holes were formed according to three designs based on size: 50 μm × 50 μm, 100 μm × 100 μm, and 200 μm × 200 μm. The ratio of a through-hole side to the space between two through-holes was designed as one to three, as shown in Fig. S1. The spaces between holes of the 50-μm, 100-μm, and 200-μm through-hole devices were 150 μm, 300 μm, and 600 μm, respectively, and these devices contained arrays of 18 × 18, 9 × 9, and 4 × 5 holes, respectively. Thus, the sums of the total through-hole areas (total DI water surface open to the air) were 0.81 mm^2^, 0.81 mm^2^, and 0.80 mm^2^, respectively. The patterned photoresist films were UV-cured to make the films rigid against the etching process. Afterwards, the backside of each Si chip was coated with photoresist. The Si chip was dipped into a HF solution to etch the SiO_2_ layer, followed by deep reactive ion etching (etching depth: 200 μm). The Si chips were then sonicated to break the remaining SiO_2_ membranes and produce the through-holes.

### NTAPPJ

The NTAPPJ system used in this study is described in previous studies. Briefly, the system consisted of a 150-mm-long glass tube tapered from an inner diameter of 4 mm to 800 μm at the orifice. Helium gas was fed into the glass tube at a fixed flow rate of 0.5 slpm. A 15-mm-long Cu external electrode was wound onto the glass tube of the NTAPPJ system at a distance of 40 mm from the end of capillary. A high-voltage bipolar square wave pulse of 7 kV_p-p_ (peak-to-peak) at 10 kHz was applied to the externally powered electrode. The glass capillary tube and powered electrode were placed in a 10-mm-thick polytetrafluoroethylene (PTFE) housing to shield the high-voltage electrode. The length of the glowing plasma jet was 10 mm under the operating conditions described above. The discharge voltage and current waveforms of the NTAPPJ were measured using a high-voltage probe (P6015A, Tektronix Inc., Beaverton, USA) and a current monitor (2877, Pearson Electronics Inc., Palo Alto, USA), respectively. Since the plasma jet flow could bring air to the plasma generation region, there was a possibility that the plasma jet flow affected the amounts and/or compositions of the RONS delivered to the water.

In the case of direct irradiation, the plasma irradiation area (plasma-irradiation water surface area) was estimated to be 0.50 mm^2^ from the orifice diameter of the NTAPPJ source (ϕ800 μm). For the indirect plasma irradiation, considering the arrangement of the through-holes in each chip, the diameter of the NTAPPJ (ϕ800 μm) covered 8–13, 2–4, and 0–1 through-holes for the 50-μm, 100-μm, and 200-μm devices, respectively (Supplementary information, Fig. S1). Thus, the maximum plasma-irradiated water surface areas were estimated to be approximately 0.04 mm^2^, 0.04 mm^2^, and 0.03 mm^2^ for the 200-μm, 100-μm, and 50-μm through-hole devices, respectively.

### *In situ* UV absorption spectroscopy

A double-beam UV-vis spectrophotometer (U-3900, Hitachi High Tech Science Co., Tokyo, Japan) was used to measure the RONS in DI water inside a quartz cuvette (100-QS, Hellma Analytics, Müllheim, Germany) with a standard optical path of 1 cm. The quartz cuvette size was 10 mm × 9.5 mm × 43 mm (inner dimensions), and its volume was 4.1 mL. A sufficiently large amount of DI water was poured into the cuvette and held near the top of the cuvette by the surface tension. We carefully placed the through-hole device on the water and pushed it from one side to the other, removing a lump of water. Thus, there was no air gap between the through-hole device and the water surface in the initial state.

The spectrophotometer detects a broad wavelength range from 190 to 900 nm. We confirmed that the absorption of the plasma-generated RONS introduced into the DI water either by direct irradiation or via the through-hole devices was below 300 nm (in the UV range), and there was no absorption in the visible and near IR range (400–900 nm). Thus, the wavelength range for the UV absorption spectroscopy was fixed at 190–340 nm. It took 90 s for the spectrophotometer to scan the wavelength range. The spectral resolution was 0.2 nm, and the scan speed was 120 nm min^−1^. The absorbance was defined by *A* = −log(*I’*/*I*), where *I’* is the transmittance of the DI water exposed to the NTAPPJ and *I* is the transmittance before the He NTAPPJ exposure. The total absorbance was calculated by integrating the absorbance^49^,^50^,^51^. Each measurement was carried out three times.

### Absolute concentration of RONS

To obtain quantitative data on the specific plasma-generated RONS in DI water, the UV absorption spectra were curve-fitted using the reference data. The fitting results were used to calculate the absolute concentrations of RONS. The reference data were experimentally obtained by measuring the UV absorption spectra of solutions that could produce the same chemical species and RONS. H_2_O_2_, NaNO_2_, and HNO_3_ solutions were used to represent the H_2_O_2_, 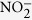, and 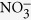 in DI water, respectively. Each solution was adjusted to concentrations of 1 to 10,000 mg L^−1^ and measured repeatedly and carefully to complete the ideal spectra over the full wavelength range. Notably, O_2_ molecules were dissolved in the water, with a concentration of 250 μM (8 mg L^−1^) at atmospheric pressure and room temperature. Using the He gas flow, the dissolved O_2_ was purged from the DI water. As the dissolved O_2_ was purged, the UV absorption spectrum showed a negative absorbance because of the increase in transmittance with the decreasing O_2_ in the DI water. The negative absorbance stopped changing after 30 min of He gas purging; we assumed that this indicated that the dissolved O_2_ was removed (0 mg L^−1^). Thus, the negative absorbance spectrum acquired under a longer He flow was used as a calibration curve for the dissolved O_2_.

### Numerical calculation of gas flow

The gas flow during the NTAPPJ irradiation was modelled as a two-dimensional flow (Supplementary information, Fig. S2). The area was 5 mm × 5 mm. Ten through-holes were prepared in the centre of the area, and each was assumed to be 100 μm in width and 200 μm in depth. The gas inlet region (width: 800 μm) was located in the centre. The symmetry of the area allowed us to use periodic boundary conditions at the centre. Consequently, half of the area was analysed, reducing the calculation time. The Navier–Stokes equation was numerically solved using the velocity-pressure method. The size of the calculation mesh was 10 μm. The boundary conditions were as follows. At the gas inflow area, the vertical velocity *v*_*y*_ was set to 1, while the horizontal velocity *v*_*x*_ was set to 0. For the pressure, the boundary condition was ∂*p*/∂*y* = 0. At the solid surface boundary, the horizontal and vertical velocities were set to 0 (*v*_*x*_ = 0, *v*_*y*_ = 0). For the pressure at the solid surface, the boundary condition was ∂*p*/∂***n*** = 0, where ***n*** is the unit vector normal to the Si chip surface. At the gas-liquid interface, the boundary conditions for the lateral and vertical velocities were *dv*_*x*_/*dy* = 0 and *v*_*y*_ = 0, respectively. For the pressure at the gas-liquid interface, the boundary condition was ∂*p*/∂*y* = 0. For the outflow area, the boundary conditions were *dv*_*x*_/*dx* = *dv*_*y*_/*dx* = *dp*/*dx* = 0.

### Water vaporisation measurements

A cuvette filled with DI water was set on a digital scale (ViBRA), and the weight change was measured as a function of time. The measurements were conducted under four conditions: direct plasma irradiation to DI water in a cuvette and indirect plasma irradiation using the three Si chips with different through-holes (200 μm × 200 μm, 100 μm × 100 μm and 50 μm × 50 μm) placed on the DI water filled cuvette.

## Additional Information

**How to cite this article**: Oh, J.-S. *et al*. Plasma cell treatment device *Plasma-on-Chip*: Monitoring plasma-generated reactive species in microwells. *Sci. Rep.* **7**, 41953; doi: 10.1038/srep41953 (2017).

**Publisher's note:** Springer Nature remains neutral with regard to jurisdictional claims in published maps and institutional affiliations.

## Supplementary Material

Supplementary Information

## Figures and Tables

**Figure 1:**
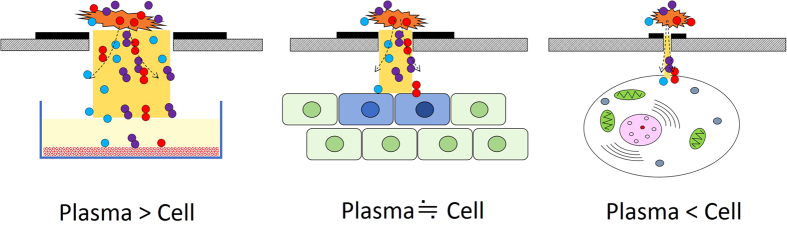
Plasma-irradiation illustrations showing the transition of the sample treatment upon reducing the size of the plasma-irradiation area. A plasma-irradiation area larger than a cell is suitable for the treatment of tissues and/or groups of cells cultured in a dish. When the plasma-irradiation area is reduced to a size as small as a cell, the cells are treated individually. When the plasma-irradiation area is reduced to smaller than a cell, specific cell functions are expected to be selectively activated or inactivated.
