# Supplementary material for: Plasma cell treatment device Plasma-on-Chip: Monitoring plasma-generated reactive species in microwells
Source: Sci Rep. 2017 Feb 8;7:41953. doi: 10.1038/srep41953 (PMC5296909; doi:10.1038/srep41953)
Supplement: Supplementary Information [file srep41953-s1.pdf]

Plasma cell treatment device *Plasma-on-Chip*:
Monitoring plasma-generated reactive species in microwells
(Supplementary Information)

Jun-Seok Oh[1,2], Shinya Kojima[3], Minoru Sasaki[3], Akimitsu Hatta[1,2*] & Shinya Kumagai[3*]

[1]Department of Electronic and Photonic Systems Engineering, Kochi University of Technology, Kami, Kochi, 782-8502 Japan. [2]Center for Nanotechnology, Research Institute of Kochi University of Technology, Kami, Kochi, 782-8502 Japan. [3]Department of Advanced Science Engineering, Toyota Technological Institute, Nagoya, 468-8511 Japan. *Corresponding authors: A. H. (email: hatta.akimitsu@kochi-tech.ac.jp) and S. Kumagai (email: kumagai.shinya@toyota-ti.ac.jp)

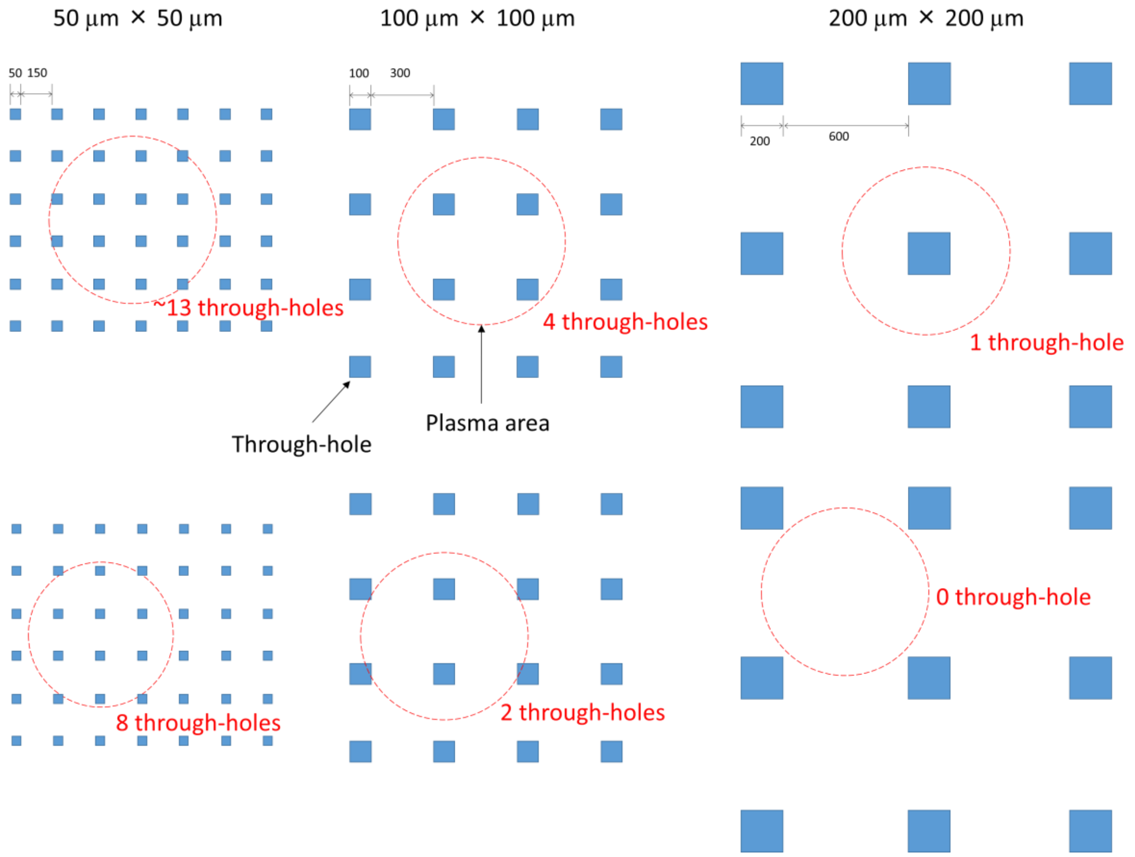

Figure S1 Schematic illustrations showing the plasma irradiation areas (circle) and through-holes (square).

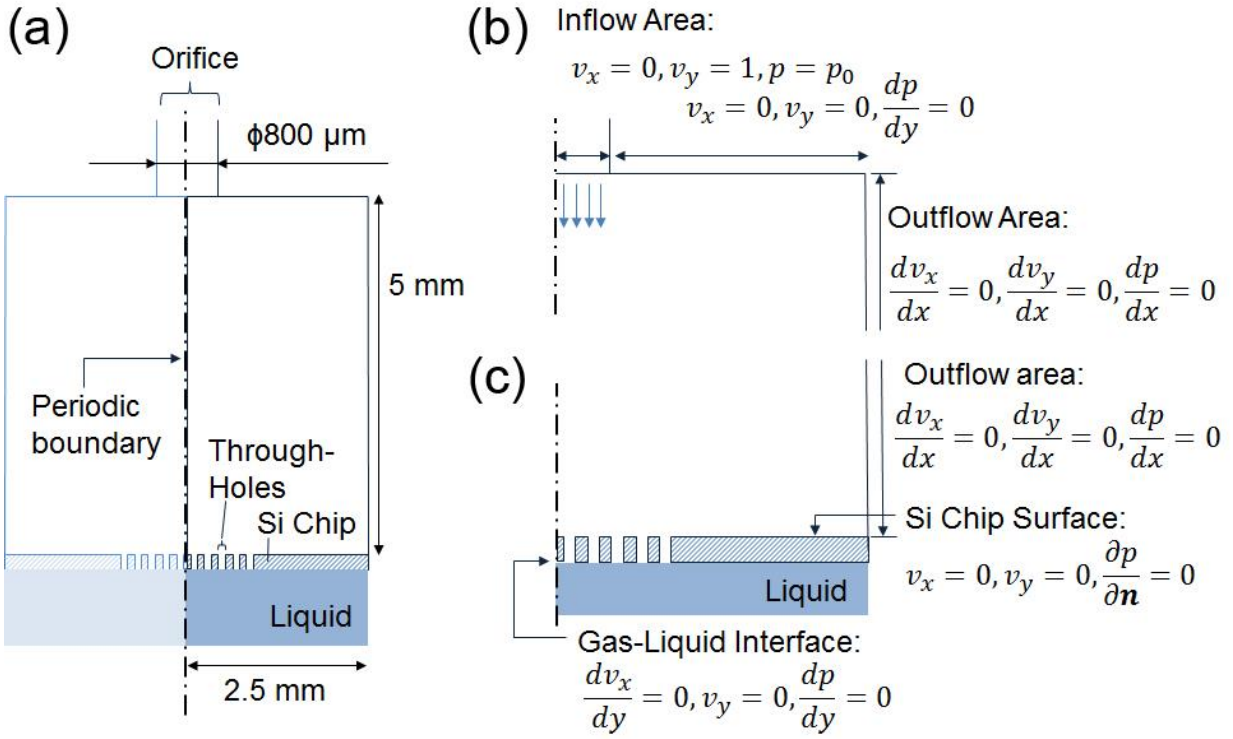

Figure S2. (a) Geometry of calculation area. A periodic boundary conditions is applied to the centre axis (indicated by the dashed line). Using the periodic boundary condition, half of the area is calculated. (b) Boundary conditions at inflow area. The vertical velocity is given at the orifice. (c) Boundary conditions in the vicinity of the through-hole device.
